# Smartphone Apps for Patients With Hematologic Malignancies: Systematic Review and Evaluation of Content

**DOI:** 10.2196/35851

**Published:** 2022-09-20

**Authors:** Nerea Báez Gutiérrez, Héctor Rodríguez Ramallo, Marcos Fernández González, Laila Abdel-Kader Martín

**Affiliations:** 1 Hospital Universitario Reina Sofía Córdoba Spain; 2 Hospital Universitario Virgen del Rocío Sevilla Spain

**Keywords:** hematological malignancies, mobile apps, smartphone, eHealth, mHealth, cancer, mobile app, mobile health, hematology

## Abstract

**Background:**

Hematological malignancies (HMs) are a heterogeneous group of cancers representing a significant cause of morbidity and mortality. The chronification of HMs and the increasing use of smartphones may lead patients to seek their current unmet needs through mobile health apps.

**Objective:**

The goal of this review was to identify and assess the quality of smartphone apps aimed at patients diagnosed with HMs.

**Methods:**

A systematic search of apps that were aimed at patients diagnosed with HMs, accessed from a Spain IP address, and were available on the iOS (App Store) and Android (Google Play) platforms was conducted in November 2021. The search terms used were “hematology,” “blood cancer,” “leukemia,” “lymphoma,” and “myeloma” apps in English, Spanish, or both languages. The identified apps were downloaded and analyzed independently by 2 reviewers. Information about general app characteristics was collected. The Mobile Application Rating Scale (MARS) was used to assess quality. The resulting parameter of the analyses, the mean score of the apps, was compared by Student *t* test.

**Results:**

Overall, 18 apps were identified; 7 were available on Android, 5 were available on iOS, and 6 were available on both platforms. All included apps were free; 3 were published in 2021, and among the apps published before 2021, only 6 were updated in 2021. Most (16/18, 89%) of the apps were aimed at patients with leukemia or lymphoma (16). The primary purposes of the apps were to provide general information about the condition (16/18, 89%) and monitor symptoms and clinical parameters (11/18, 61%). Health care professionals contributed to the development of 50% (9/18) of apps; 6 were owned and supported by scientific societies, and 3 were developed with the participation of health care professionals. The mean MARS score for the overall quality of the apps was 3.1 (SD 1.0). The engagement and aesthetics subscales were the lowest rated subscales, with only 44% (8/18) and 67% (12/18), respectively, of the apps obtaining acceptable scores. None of the included apps proved clinical efficacy through clinical trials in patients with HMs. Statistically significant differences were found in the MARS scores between operating systems (+1.0, *P*=.003) in favor of iOS apps. The participation of health care professionals in the development of the apps did not have a statistically significant impact on the MARS scores.

**Conclusions:**

This systematic search and evaluation identified few acceptable quality mobile apps for patients with HMs. Current and future apps for patients with HMs should provide evidence-based valuable information, improve user engagement, incorporate functions according to patient preferences, and generate evidence regarding the efficacy of app use by patients with HMs.

## Introduction

Hematological malignancies (HMs) are a heterogeneous group of cancers that affect hematopoietic and lymphoid tissue. These disorders constitute 6.8% of all cancers, representing a significant cause of morbidity and mortality [1]. In the past decade, an increased incidence rate of HMs has been described [2,3], possibly due to a growth in life expectancy and the aging of the population [4].

HMs are frequently aggressive and require urgent, lengthy, and burdensome treatment. The complexity of HM treatments could reduce patient adherence and increase the risk of potential drug-drug interactions and adverse events [5-10]. Furthermore, patients with HMs and, specifically, patients with lymphoma and myeloma often experience psychological despair and poor quality of life throughout their illness course [11-13]. Although HM treatments add a burden to various aspects of a patient’s life, the wide range of treatments now available has significantly improved the management of these patients, often transforming HMs into chronic diseases with long-term survival [14,15].

The chronification of HMs in the digital era may lead patients to seek their current unmet needs through internet-based health care, mobile health (mHealth), and, specifically, smartphone apps. An increase in mHealth use is a likely scenario, since the use of smartphones is growing, with estimates indicating that 77.6% of Europeans own a smartphone [16]. In this context, health apps designed for patients with HMs could serve as additional tools for telemedicine, patient education and life coaching, medication adherence, communication, and social media connections [17-19].

As the number of health-related apps has increased rapidly in the last decade, there is an increasing need for research to assess the quality of eHealth tools and identify patients’ unmet needs.

This study aimed to identify apps that are accessible from Spain and are designed for patients diagnosed with HMs, analyze their characteristics, and evaluate their quality with the Mobile Application Rating Scale (MARS) [20].

## Methods

### Search Strategy

A systematic search of apps that were aimed at patients diagnosed with HMs, accessed from a Spain IP address, and were available on the iOS (App Store) and Android (Google Play) platforms was conducted from November 10, 2021, to November 25, 2021. The search was carried out following the PRISMA (Preferred Reporting Items for a Systematic Review and Meta-analysis) guidelines [21].

The search terms used were “hematology,” “blood cancer,” “leukemia,” “lymphoma,” “myeloma,” and their equivalents in the Spanish language—“hematología,” “cáncer de Sangre,” “leucemia,” “linfoma,” and “mieloma,” respectively.

The inclusion criteria used for selection were apps available in English or Spanish that were targeted at patients with HMs and included general information about HMs. The exclusion criteria used were apps that included inappropriate content (including horoscopes and astrology, among others), apps exclusively for fundraising for HMs that did not provide functions or tools designed for their use, or apps with nonfunctional links. The apps analyzed had to comply with all inclusion criteria to be included in the data extraction phase.

The names and descriptions of the apps from the search on Google Play and the App Store were selected against a priori selection criteria. The apps that met the inclusion criteria were downloaded for further screening using an Mi 9 Lite smartphone (Android version 10, Xiaomi Inc) for Google Play apps. For the apps downloaded from the App Store, an iPad Pro 2020 (Apple Inc) running iPadOS 15.1 was used. For apps duplicated across stores, the iOS app was evaluated because of the lower number of apps in the App Store compared with those in Google Play.

### Data Extraction

Data were obtained from the App Store and Google Play online app descriptions (app characteristics and the narrative text) by 2 independent researchers. Data were extracted and entered into a structured Excel database (Microsoft Corp).

The variables collected for each app were the name, developer/owner name, type of developer/owner (commercial, scientific society, patient association), platform (Android or iOS), language (English or Spanish), app store category (eg, education, health and fitness, medicine), cost (€), date of publication (year), date of the last update (year), app file size (MB), app version, participation of health care professionals in the design and development of the app (yes/no), and HM targeted (eg, acute lymphocytic leukemia, multiple myeloma, Hodgkin lymphoma).

The participation of health care professionals was considered when specified in the app description or when the app was developed by a health organization (eg, a scientific society or a hospital). The purposes of the apps were classified into the following categories: assessment (eg, providing clinical scales, classifying adverse event severity, or interpreting laboratory findings), general information (eg, information about HMs, medications, or adverse events), the monitoring of clinical parameters (eg, register of laboratory parameters or symptoms), register of patient activities (eg, calendars for patients to add appointments and treatment administration), and contact with health care professionals or other patients.

A descriptive analysis was developed with continuous and discrete variables presented as mean and standard deviation and frequency and percentage, respectively. The means of continuous variables were compared using a *t* test. Results with a *P* value of <.05 were considered statistically significant. The generated data were analyzed using SPSS (version 26.0, IBM Corp).

### App Quality Evaluation

To evaluate app quality, the MARS tool was used [20]. The MARS is a validated system to assess health apps that has been described as the most comprehensive for evaluating technical information and capabilities of apps [20,22,23]. This tool has been widely used to evaluate health apps designed for many diseases [24-30].

The MARS comprises 23 items across 5 subscales:

- Engagement: evaluates the entertainment, interest, customization, interactivity, and adequacy of the target group
- Functionality: assesses the performance, ease of use, navigation, and gestural design of the app
- Aesthetics: examines the layout, graphics, and visual appeal of the app
- Information: assesses the accuracy of the description, establishment of goals, quality and quantity of information, visual information, credibility of the source, and evidence-based development of the app
- Subjective quality: determines willingness to recommend app, times app will be used, willingness to pay, and overall rating of app

Each criterion is evaluated from 1 to 5 (1=inadequate; 2=poor; 3=acceptable; 4=good; 5=excellent). A mean score of the 5 subscales is calculated to describe overall quality.

Before the app search was carried out, reviewers read and became acquainted with the MARS tool. All authors then discussed each rating criteria to achieve a consensus on how to apply them. The first app included was evaluated concurrently by all reviewers to ensure a common understanding and application of the MARS tool. The apps included were independently assessed by 2 reviewers, whose scores were then compared, and a final MARS score was agreed upon. A third reviewer was invited to resolve discrepancies if a consensus on the final MARS score was not reached.

In order to quantify the subjectivity degree of the reviewers’ evaluations, the interrater reliability of the quality scores between the independent evaluations was calculated using the intraclass correlation coefficient (ICC) for a 2-way random effects model.

## Results

### Overview

**Figure 1:**
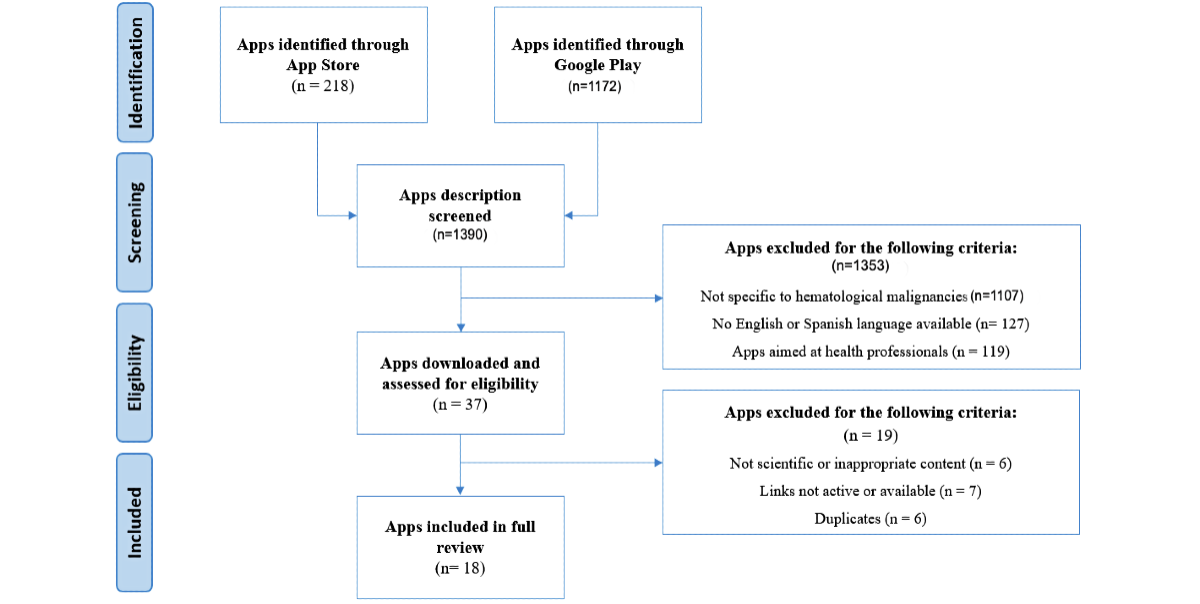
Prisma Flow-Chart.

The titles, descriptions, and screenshots of 1390 apps were screened in a pair-review fashion. Overall, 18 apps met the inclusion criteria for a comprehensive evaluation with the MARS tool (Figure 1).

### App Characteristics

Table 1 displays the general features of the apps. Regarding the apps available on the platforms searched, 7 were available on Google Play (Android), 5 were available on the App Store (iOS), and 6 were available on both platforms. The apps were categorized in different sections in their respective platforms, as follows: medicine (11), health and well-being (4), education (1), news (1), and books and reference works (1). All apps evaluated were free. The average file size of the apps was 27.2 (SD 25.3) MB.

Table 2 displays apps’ purposes and health care professional involvement in their design. All 7 apps with a unique purpose were designed to provide information about HMs and their management. Eleven apps had multiple purposes, the most frequent being providing information and monitoring symptoms and clinical parameters. Overall, 9 of the apps were developed with the participation of health care professionals, 6 were owned and supported by scientific societies, and 3 were developed with the participation of physicians or nurses. None of the included apps reported clinical efficacy through clinical trials in patients with HMs.

**Table 1:** Smartphone app technical characteristics.

Name	Year of publication	Year of the last update	Platform			Language	
			Android	iOS	English		Spanish
ALL Manager	2019	2021		✓	✓		
CLL Manager	2017	2021		✓	✓		
CLL Watch and Wait Tracker	2017	2020	✓	✓	✓		
CML Life	2018	2019	✓	✓	✓		✓
CML Today	2015	2015	✓	✓	✓		✓
Don’t Walk Alone	2017	2018		✓	✓		
Focus on Lymphoma	2013	2018	✓		✓		
Hodgkin Lymphoma Manager	2019	2021		✓	✓		
Leucemia: Síntomas Y Tratamiento: FAQ	2018	2018	✓		✓		
Leukaemia (Leukaemia) News	2020	2020	✓		✓		
Leukemia: Causes, Diagnosis, and Treatment	2021	2021	✓		✓		
Leukemia Disease	2017	2017	✓		✓		✓
Leukemia Drug Tracker	2021	2021	✓		✓		
LLS CAR T	2020	2020	✓	✓	✓		
LLS Health Manager	2019	2021	✓	✓	✓		
LRF Understanding Lymphoma	2020	2021	✓	✓	✓		
Mieloma	2021	2021	✓				✓
Multiple Myeloma Manager	2015	2021		✓	✓		

**Table 2:** App purpose and health care professional involvement.

Name	Hematologic malignancy	Purpose						HPP^a^
		I^b^	M^c^	R^d^	C^e,f^	CP^g^		
ALL Manager	ALL^h^	✓	✓	✓	✓	✓		
CLL Manager	CLL^i^	✓	✓	✓	✓	✓		
CLL Watch and Wait Tracker	CLL	✓	✓				✓	
CML Life	CML^j^	✓	✓				✓	
CML Today	CML		✓	✓			✓	
Don’t Walk Alone	CLL	✓	✓		✓	✓	✓	
Focus on Lymphoma	Lymphoma	✓	✓	✓		✓	✓	
Hodgkin Lymphoma Manager	HL^k^	✓	✓	✓	✓	✓		
Leucemia: Síntomas Y Tratamiento: FAQ	Leukemia	✓						
Leukaemia (Leukaemia) News	Leukemia	✓						
Leukemia: Causes, Diagnosis, and Treatment	Leukemia	✓						
Leukemia Disease	Leukemia	✓						
Leukemia Drug Tracker	Leukemia	✓	✓	✓				
LLS CAR T	HM^l^	✓					✓	
LLS Health Manager	HM	✓	✓	✓	✓	✓	✓	
LRF Understanding Lymphoma	Lymphoma	✓					✓	
Mieloma	MM^m^	✓					✓	
Multiple Myeloma Manager	MM	✓	✓	✓	✓	✓		

^a^HPP: health care professional participation.

^b^I: general information.

^c^M: monitoring of symptoms and clinical parameters.

^d^R: register of patient activities.

^e^C: contact with health care professionals.

^f^This feature was included in the app but was not available for Spanish patients.

^g^CP: contact with other patients.

^h^ALL: acute lymphocytic leukemia.

^i^CLL: chronic lymphocytic leukemia.

^j^CML: chronic myeloid leukemia.

^k^HL: Hodgkin lymphoma.

^l^HM: hematological malignancy.

^m^MM: multiple myeloma.

### App Quality Assessment

Tables 3 and 4 display the MARS subscales scores for the individual apps. The scores in the functionality section were similar between the apps. The movements between menus and screens were fast and satisfactory for most apps. The most remarkable differences were found in the information and aesthetics sections, for which only 67% (12/18) and 61% (11/18) of the apps showed acceptable scores (>3), respectively. None of the apps were tested in clinical trials.

The mean MARS score was 3.1 (SD 1.0), considering this value as acceptable. Table 5 contains the overall quality scores. For 5 apps, the mean MARS score was >4, meaning these apps were of good quality. On the other hand, 7 apps received a mean score of <3 and were classified as poor-quality apps.

iOS apps obtained better MARS scores when compared to apps for Android (+1.0) points when comparing apps based on their platforms. This difference was statistically significant for the mean MARS score (*P*=.003) and for all 5 domains: engagement (+1.0; *P*=.001), functionality (+0.6, *P*=.05), aesthetics (+1.1, *P*=.01), information (+1.0, *P*=.01), and subjective evaluation (+1.1, *P*=.002). For apps available on both platforms, the MARS mean scores were higher than those for apps only available on one of the platforms (+0.6, *P*=.19). The aesthetic domain score was statistically superior for apps available on both platforms (+1.2, *P*=.04). The participation of health care professionals had a positive impact on the appraisals of the apps (+0.6 points), but no statistically significant differences were found (*P*=.22).

For the overall MARS ratings, the ICC was 0.94 (95% CI 0.75-0.98), confirming excellent interrater reliability. For the engagement, functionality, aesthetics, information, and subjective domains, the ICCs were 0.84, 0.86, 0.94, 0.95, and 0.93, respectively.

**Table 3:** The Mobile Application Rating Scale engagement and functionality subscales.

Name	Engagement, score						Functionality, score			
	Ent^a^	Int^b^	Cus^c^	Iy^d^	Tg^e^	Per^f^		EU^g^	Nav^h^	GD^i^
ALL Manager	4	4	4	4	4	4		4	4	4
CLL Manager	4	4	4	4	4	4		4	4	4
CLL Watch and Wait Tracker	2	3	3	2	4	5		3	4	4
CML Life	4	4	4	2	4	3		3	4	4
CML Today	2	3	3	2	4	5		4	4	4
Don’t Walk Alone	2	3	3	3	4	3		3	3	3
Focus on Lymphoma	4	4	4	3	5	4		5	4	5
Hodgkin Lymphoma Manager	4	4	4	4	4	4		4	4	4
Leucemia: Síntomas Y Tratamiento: FAQ	2	2	1	1	3	3		3	3	3
Leukaemia (Leukaemia) News	2	2	1	1	3	2		4	2	2
Leukemia: Causes, Diagnosis, and Treatment	2	1	1	1	1	2		2	1	1
Leukemia Disease	2	2	1	1	3	2		3	3	3
Leukemia Drug Tracker	2	1	2	2	3	2		3	3	3
LLS CAR T	4	4	1	1	4	5		4	5	5
LLS Health Manager	4	3	3	4	4	4		4	4	4
LRF Understanding Lymphoma	4	4	1	1	4	4		3	4	3
Mieloma	1	2	2	3	2	3		2	4	3
Multiple Myeloma Manager	4	4	4	4	4	4		4	4	4
Overall, mean (SD)	2.9 (1.1)	3 (1.1)	2.6 (1.3)	2.4 (1.2)	3.6 (0.9)	3.5 (1.0)		3.4 (0.8)	3.6 (0.9)	3.5 (1.1)

^a^Ent: entertainment.

^b^Int: interest.

^c^Cus: customization.

^d^Iy: interactivity.

^e^Tg: target group.

^f^Per: performance.

^g^EU: ease of use.

^h^Nav: navigation.

^i^GD: gestural design.

**Table 4:** The Mobile Application Rating Scale aesthetics and information subscales.

Name	Aesthetics, score				Information, score					
	Lay^a^	Gra^b^	VA^c^	AAD^d^		Goa^e^	QI^f^	QyI^g^	VI^h^	Cre^i^
ALL Manager	4	4	4	5		3	5	5	4	3
CLL Manager	4	4	4	5		3	5	5	4	3
CLL Watch and Wait Tracker	4	3	4	5		N/A^j^	4	3	N/A	3
CML Life	4	3	4	4		N/A	3	3	4	3
CML Today	4	3	3	4		4	N/A	N/A	4	3
Don’t Walk Alone	5	4	4	3		N/A	2	4	2	2
Focus on Lymphoma	5	4	4	5		N/A	5	5	4	3
Hodgkin Lymphoma Manager	4	4	4	5		3	5	5	4	3
Leucemia: Síntomas Y Tratamiento: FAQ	1	2	1	1		N/A	2	1	N/A	1
Leukaemia (Leukaemia) News	1	2	1	4		N/A	1	3	N/A	2
Leukemia: Causes, Diagnosis, and Treatment	1	2	1	2		N/A	1	2	N/A	1
Leukemia Disease	1	2	1	1		N/A	2	2	N/A	1
Leukemia Drug Tracker	2	1	2	4		N/A	3	1	N/A	1
LLS CAR T	5	5	4	5		N/A	4	4	5	3
LLS Health Manager	4	3	4	5		4	4	3	4	3
LRF Understanding Lymphoma	4	5	5	5		N/A	5	4	4	3
Mieloma	3	1	2	4		N/A	3	3	N/A	3
Multiple Myeloma Manager	4	4	4	5		3	5	5	4	3
Overall, mean (SD)	3.3 (1.5)	3.1 (1.2)	3.1 (1.4)	4 (1.4)		3.3 (0.5)	3.5 (1.5)	3.4 (1.4)	4 (0.7)	2.4 (0.8)

^a^Lay: layouts.

^b^Gra: graphics.

^c^VA: visual appeal.

^d^AAD: accuracy of app description.

^e^Goa: goals.

^f^QI: quality of information.

^g^QyI: quantity of information.

^h^VI: visual information.

^i^Cre: credibility.

^j^N/A: not applicable.

**Table 5:** The Mobile Application Rating Scale overall quality scores.

Name	Engagement (score), mean (SD)	Functionality (score), mean (SD)	Aesthetics (score), mean (SD)	Information (score), mean (SD)	Subjective quality (score), mean (SD)	Overall score, mean (SD)
ALL Manager	4 (0.0)	4 (0.2)	4 (0.0)	4.2 (1.0)	4.3 (0.8)	4.1 (0.1)
CLL Manager	4 (0.0)	4 (0.2)	4 (0.0)	4.2 (1.0)	4.3 (0.8)	4.1 (0.1)
CLL Watch and Wait Tracker	2.8 (0.8)	4 (0.7)	3.7 (0.5)	3.8 (1.0)	2.8 (0.4)	3.4 (0.6)
CML Life	3.6 (0.9)	3.5 (0.5)	3.7 (0.5)	3.5 (0.6)	2.4 (0.8)	3.3 (0.5)
CML Today	2.8 (0.8)	4.3 (0.4)	3.3 (0.5)	3.8 (0.5)	2.5 (0.9)	3.3 (0.7)
Don’t Walk Alone	3 (0.7)	3 (0.0)	2.8 (0.2)	2.6 (0.9)	1.8 (0.4)	2.7 (0.5)
Focus on Lymphoma	4 (0.7)	4.6 (0.5)	4.3 (0.5)	4.4 (0.9)	3.6 (0.5)	4.2 (0.4)
Hodgkin Lymphoma Manager	4 (0.0)	4 (0.2)	4 (0.0)	4.2 (1.0)	4.3 (0.8)	4.1 (0.1)
Leucemia: Síntomas Y Tratamiento: FAQ	1.8 (0.8)	3 (0.0)	1.3 (0.5)	1.3 (0.5)	1.5 (0.4)	1.8 (0.7)
Leukaemia (Leukaemia) News	1.8 (0.8)	2.5 (0.9)	1.3 (0.5)	2.5 (1.3)	1.8 (0.4)	2.0 (0.5)
Leukemia: Causes, Diagnosis, and Treatment	1.2 (0.5)	1.5 (0.5)	1.3 (0.5)	1.5 (0.6)	1.1 (0.2)	1.3 (0.2)
Leukemia Disease	1.8 (0.8)	2.8 (0.4)	1.3 (0.5)	1.5 (0.6)	1.3 (0.3)	1.8 (0.6)
Leukemia Drug Tracker	2 (0.7)	2.8 (0.4)	1.7 (0.5)	2.3 (1.5)	1.5 (0.4)	2.0 (0.5)
LLS CAR T	2.8 (1.7)	4.8 (0.4)	4.7 (0.5)	4.2 (0.8)	2.3 (0.8)	3.8 (1.1)
LLS Health Manager	3.6 (0.5)	4 (0.0)	3.7 (0.5)	3.8 (0.8)	2.5 (0.4)	3.5 (0.6)
LRF Understanding Lymphoma	2.8 (1.7)	3.5 (0.5)	4.7 (0.5)	4.2 (0.8)	3.1 (0.7)	3.7 (0.8)
Mieloma	2.0 (0.7)	3 (0.7)	2 (0.8)	3.3 (0.5)	1.7 (0.4)	2.4 (0.7)
Multiple Myeloma Manager	4 (0.0)	4 (0.2)	4 (0.0)	4.2 (1.0)	4.3 (0.8)	4.1 (0.1)
Overall	2.9 (0.9)	3.5 (0.8)	3.1 (1.3)	3.3 (1.1)	2.6 (1.1)	3.1 (1.0)

## Discussion

### Principal Findings

Health apps have the potential to become a standard of care for chronic patients. Apps have been associated with positive patient outcomes such as improved adherence to medication and quality of life and decreased use of health care–related resources [31]. Therefore, health apps may offer valuable tools for patients with HMs in a health care context [17,32-34].

This systematic review highlighted the limited number of apps available for patients with HMs. The number of apps identified in this study was significantly lower than previously published reviews on COVID-19, neoplasms, and specific types of cancer such as breast cancer [35-37]. The apps were developed for a narrow range of diseases that HMs encompass. Currently, patients with acute myeloid leukemia or non-Hodgkin lymphoma do not have access to eHealth tools in the main app stores that specifically target these diseases. In addition, there is a lack of apps specifically designed for children, regardless of them being a significant population of patients with HMs who have distinct needs [38,39].

The low number of apps found in this review may be due to several reasons. First, HMs are relatively rare; according to the Global Cancer Observatory, HMs account for an estimated 6.8% of all cancers worldwide [1]. Similarly, systematic searches for diseases with low prevalence tend to find few apps directed to patients [1,24,40,41]. Second, strict exclusion criteria were applied for app selection—specifically, the exclusion of apps aimed at health care professionals, which may encompass half of the health apps [42]. Third, HMs often affect older patients, an age group for which developers may often not design eHealth tools [43,44]. Older age has been associated with a lack of interest in eHealth and smartphones [45,46]. This population also faces additional barriers to using eHealth, such as physical disability and technology inexperience [47,48].

All apps included for evaluation were designed to serve as an information source or monitoring tool for medications and symptoms. The information was not frequently reviewed and updated, as only half of the HM apps (9/18, 50%) were updated during 2021. The management of HMs is in continuous change, often requiring guidelines and recommendations to be updated with frequency, often yearly. As an illustration of this constant evolution, the European Medicines Agency has recommended for approval an average of 5 drugs for HMs per year [49]. The growing arsenal of new treatments highlights the importance of regularly updating apps according to changes in guidelines and recommendations, as the management of HMs is a complex field of constant change [50].

Among the few apps that complied with the inclusion criteria for this study, the overall quality was acceptable. The highest rated subscale on the MARS was functionality due to app efficiency and ease of use. On the other hand, the engagement subscale was the worst evaluated, specifically the customization and interactivity items. Poor results in the engagement domain could mean that even if quality apps include valuable tools, patients will use them for limited periods. Furthermore, the customization of app interactions by setting personalized reminders is perceived as highly beneficial by patients with HMs [51-53]. Finally, the credibility item was poorly evaluated, as the existence of commercial interests or the source’s legitimacy could not be verified.

Currently, there is a lack of high-quality evidence on the eHealth needs of patients with HMs. Surveys about communication technologies conducted on hematology and oncology patients found that mobile phones were the most frequently used device to search for health-related information. Patients were highly interested in staying informed about health issues, disease prevention, healthy lifestyles, and general information about the disease [31,54]. Patients seem to be interested in eHealth helping with practical issues, such as appointment management, the provision of advice on disease and symptom management, and direct communication with health care professionals in addition to providing information. Patients were less interested in features that can add additional burdens to their daily activities, such as recording and monitoring medication, symptoms, and adverse events [31]. Patients with HMs also desire the creation of communication channels that allow health care professionals to answer their concerns rapidly [31]. A scoping review of virtual care in patients with HMs described high patient satisfaction with telemedicine interventions that allowed clinicians and nurses to communicate via phone calls or videoconferencing [33].

The 18 apps that were designed for patients with HMs and were evaluated in this review did not fulfill the previously described preferences. Although 17 apps provided information about the disease, most of the information did not focus on disease prevention, symptom management, or healthy lifestyles. In addition, direct communication with health care professionals for Spanish patients was not possible, and the management of medical appointments was not an included feature. Further research on the virtual care needs of patients with HMs is warranted; surveys and validated questionnaires explicitly designed for patients with HMs are needed to gain knowledge of the unmet needs of these patients. Understanding patient preferences through a continuous user-centered design is essential for the success of mHealth for patients with HMs [50,55]. The differences found between features included in apps and the preferences of patients with HMs highlight the apparent necessity of their participation in the design of health-related apps. The inclusion of health care professionals may not be sufficient to adequately provide quality apps that cover patient needs, as there is a divide between the expectations and needs of patients and those identified by health care professionals [56,57].

Among the stakeholders involved in the management of HMs, the participation of health care professionals in the development of mHealth apps is frequently described as a critical factor related to their general quality and, specifically, the quality of the information provided [24,25,58]. A systematic review that evaluated expert participation and adherence to medical evidence in mHealth apps showed that the lack of participation of health care professionals and inclusion of evidence-based information were persistent characteristics of the available health apps [58]. However, even when these factors are potentially related, expert involvement in the design does not guarantee adherence to evidence-based content [58,59]. In this review, half of the apps (9/18, 50%) targeting patients with HMs included the participation of health care professionals in the development phase. Nevertheless, we did not find statistically significant differences in the quality scores based on this participation. Furthermore, a cross-sectional survey of oncology and hematology patients found that more than 80% of patients would use an app if a health professional recommended it [60]. These data suggest that health care professionals may also play a crucial role in high-quality apps reaching patients with HMs.

Due to the complex nature of HMs, which often require highly complex and burdensome therapeutic protocols, we could presume that patients would benefit from additional tools that help manage different aspects of the disease. However, the role of apps in the care of patients with HMs is uncertain; a review exploring the research on eHealth intervention in HMs found that few trials were designed exclusively for patients with HMs. These studies generally included mixed cancer groups composed of a small sample of hematology patients; moreover, the principal study results were not presented separately [34]. Similarly, among the 18 apps evaluated in this review, none assessed the clinical impact of the intervention through clinical trials; this finding is common among studies that evaluate health apps, with authors often stating the need for studies that assess the efficacy of health apps [23-25,37,61,62]. Additional randomized controlled trials testing the efficacy of apps are warranted before implementing this technology for managing HMs, as eHealth may also increase the burden of treatment on vulnerable patients [63,64].

### Recommendations and Future Research for HM App Development

Current and future apps for patients with HMs should focus on improving the lowest rated subscales of the MARS scale: engagement and aesthetics. Specifically, improvements are needed in the interactivity, customization, and entertainment features, as well as in graphical design and visual appeal. Further refinement of the information provided may be needed in order to deliver updated evidence-based information adapted to the necessities and preferences of patients with HMs. In this context, HM apps should be developed through a user-centered, collaborative design process with the participation of all stakeholders involved. The participation of health care professionals can increase the credibility of apps and trustworthiness of sources [24,25,58]. Furthermore, the participation of patients with HMs is essential for developing apps that fulfill their unmet needs, increasing their usability [56,57]. App developers and stakeholders should work to include apps in specialized and primary care assistance circuits by adding functions that facilitate the access of patients with HMs to health assistance through the management of appointments and direct communication with health care professionals [31,54]. Regular updates of app contents should be performed to incorporate information that is consistent with recently generated evidence.

Future research in the field of HM apps should focus on gaining knowledge regarding the eHealth needs of patients with HMs, conducting surveys that investigate the changing needs of specific groups of patients with HMs, measuring the existence of health app safety concerns, and generating evidence-based data on the effectiveness of eHealth interventions through apps in patients with HMs.

### Limitations

This systematic review has several limitations. First, the reviewers assigned a score for each category using brief descriptions of what makes an excellent, regular, or mediocre app. Thus, there was a degree of subjectivity when evaluating apps using the MARS [21,22]. However, the multiple reviewer design of this study may narrow the impact of this limitation because of the high degree of interreviewer agreement observed. Second, despite the MARS being a validated tool that is widely used to assess the quality of apps for many diseases [23-29], it does not consider aspects such as privacy, security, and the frequency of updates to apps, which have been considered aspects of particular relevance when evaluating the quality of health software [65]. Third, as health care professionals, the authors evaluated apps aimed at patients; this has limitations, as we may not fully comprehend their needs and preferences [56,57]. Fourth, the search was limited to 2 app platforms: Google Play and the App Store. Although these platforms encompass the vast majority of apps available on the market [66], several platforms (Windows Phone, Blackberry Market, etc) were not included in this review due to the lack of availability of devices for performing the search among the reviewers. Therefore, the possibility exists that some apps dedicated to HMs were missed.

### Conclusion

The potential for eHealth to improve the care of patients with HMs exists. However, current apps need further refinement in order to provide evidence-based valuable information, keep users engaged, and provide a visually appealing interface. Moreover, there is a need to generate evidence on the efficacy of eHealth interventions in patients with HMs.

Future eHealth tools for patients with HMs should consider including all stakeholders in the design phase. Notably, the participation of patients in this phase will serve to design and implement tools according to their currently unmet needs.

## Abbreviations

HM: hematological malignancy
ICC: intraclass correlation coefficient
MARS: Mobile Application Rating Scale
mHealth: mobile health
PRISMA: Preferred Reporting Items for a Systematic Review and Meta-analysis
