# Correction: Smartphone Apps for Patients With Hematologic Malignancies: Systematic Review and Evaluation of Content

**DOI:** 10.2196/43412

**Published:** 2022-10-13

**Authors:** Nerea Báez Gutiérrez, Héctor Rodríguez Ramallo, Marcos Fernández González, Laila Abdel-Kader Martín

**Affiliations:** 1 Hospital Universitario Reina Sofía Córdoba Spain; 2 Hospital Universitario Virgen del Rocío Sevilla Spain

In “Smartphone Apps for Patients With Hematologic Malignancies: Systematic Review and Evaluation of Content” (JMIR Mhealth Uhealth 2022;10(9):e35851), the authors noted one error.

In the originally published article, the article type appeared inadvertently as “Review.”

In the corrected version, the article type appears correctly as “Original Paper.”

The correction will appear in the online version of the paper on the JMIR Publications website on October 13, 2022, together with the publication of this correction notice. Because this was made after submission to PubMed, PubMed Central, and other full-text repositories, the corrected article has also been resubmitted to those repositories.

